# The Perth Declaration to Eliminate Rheumatic Fever and Rheumatic Heart Disease: Adopted at the Second World Congress on Rheumatic Heart Disease Perth, Australia, 11–14 November 2026

**DOI:** 10.5334/gh.1605

**Published:** 2026-11-13

**Authors:** Jagat Narula, Garry Jennings, Derek P. Chew, on behalf of the Perth Declaration Writing Group

**Affiliations:** 1 World Heart Federation, Co-Chair, CH; 2 National Heart Foundation of Australia, Co-Chair, AU; 3 National Heart Foundation of Australia, AU

**Keywords:** rheumatic heart disease, acute rheumatic fever, Group A Streptococcus, elimination, Indigenous health, health equity

## Abstract

About 55 million people live with rheumatic heart disease and more than 370,000 die from it each year, almost entirely in low- and middle-income countries and among Indigenous peoples in wealthy ones. The disease is preventable, detectable, controllable and treatable with tools that have existed for decades; its persistence reflects a failure of delivery and equity, not of knowledge. The Perth Declaration, adopted at the Second World Congress on Rheumatic Heart Disease in November 2026, is issued by people living with the disease, civil society, clinicians and researchers, with governments invited to endorse it. It sets out eight commitments the community makes about its own conduct, each anchored to a Congress workshop and reported against in 2029; specific asks of governments, health services and global partners, including sustained financing, environmental health, community-controlled delivery and Indigenous data sovereignty; and phased targets to 2040, including a Group A streptococcal vaccine.

## Graphical Abstract

**Figure:**
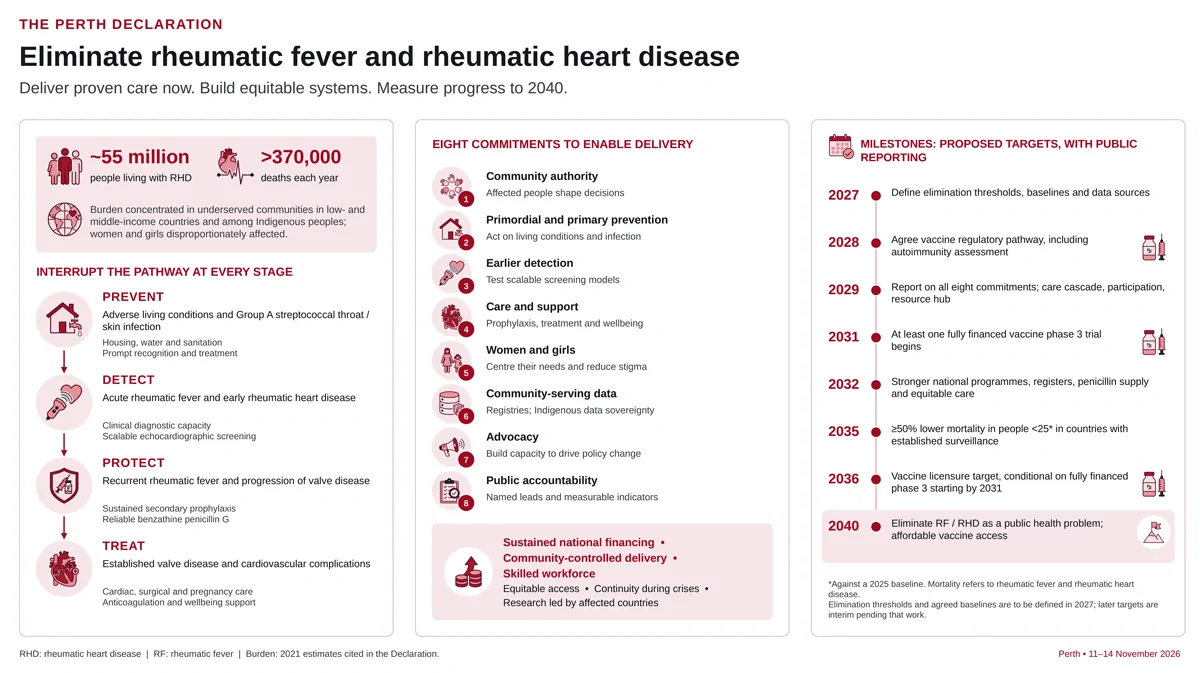

The Perth Declaration has been developed and announced on the traditional lands of the Whadjuk people of the Noongar nation. We acknowledge their continuing connection to land, water and community, and express our deep respect for their role in caring for Country. We pay respect to the ancestors of the Whadjuk people, their Elders past and present, and all Aboriginal and Torres Strait Islander people as First Nations people of Australia.

## Who We Are

We, people living with rheumatic heart disease, civil society advocates, representatives of affected communities, including First Nations and Indigenous communities, clinicians, researchers, patient organisations, the World Health Organization, and other United Nations agencies, met in Perth, on Whadjuk Noongar Boodja, for the Second World Congress on Rheumatic Heart Disease.

This Declaration is adopted by resolution of the Congress in plenary. It is signed by the Co-Chairs and the Writing Group on behalf of the Congress, and by individuals and organisations through a signature roll that remains open after the Congress closes. Ministers, parliamentarians, multilateral leaders, and institutions may endorse it at any time, whether or not they were able to join us in Perth.

## Where We Stand

Approximately 55 million people live with rheumatic heart disease, and more than 370,000 lives are lost to it each year (1,2).^1^ It is the most common acquired heart disease in people under 25, and one of the few cardiovascular conditions that affects adolescents and young adults in substantial numbers during the years in which they are completing their education, forming families, and building their working lives. Women and girls are affected in greater numbers than men and boys, and their needs have too often been overlooked.

This is not a disease distributed at random. There is a substantial burden of disease in low- and middle-income countries, and within those countries, it concentrates among the poorest communities. It persists where primary health care is thin, where diagnosis comes late, where penicillin supply is unreliable, and where cardiac surgery is out of reach.

Rheumatic heart disease also persists in high-income countries, almost entirely among Indigenous peoples and other communities living with the consequences of colonisation, dispossession, poverty, household crowding and limited access to health care. In Australia, Aboriginal and Torres Strait Islander people account for the overwhelming majority of cases. The same pattern is found among Indigenous peoples in Aotearoa New Zealand, across the Pacific, and elsewhere. These are not incidental exceptions to the global picture. They are the clearest demonstration of what drives this disease: not the wealth of a nation but living conditions, equitable access to services, and the availability of timely healthcare.

Rheumatic heart disease is therefore a measure of equity. Wherever it is found, it marks a population that has been underserved.

‘We know what needs to be done, and we know that it can be done.’

Pat Turner AM and Dr Dawn Casey, National Aboriginal Community Controlled Health Organisation (NACCHO), Australia.

## What We Affirm

Rheumatic heart disease and its precursor, acute rheumatic fever, are largely preventable through improved living conditions and environmental health, and through ready access to primary health care that promptly finds and treats Group A streptococcal throat and skin infection. They are detectable through clinical vigilance and echocardiography. They are controllable through sustained, patient-centred secondary prophylaxis that prevents recurrence and disease progression. And they are treatable through timely access to essential cardiac care, integrated into health coverage.

We affirm that the knowledge required to act already exists. What is missing is not discovery but delivery: sustained investment, political commitment, and the transfer of authority and resources to the communities most affected.

We reaffirm Sustainable Development Goal Target 3.4 on premature mortality from noncommunicable diseases (3), and the 2018 World Health Assembly resolution on rheumatic fever and rheumatic heart disease (4), and we recognise the World Congress on Rheumatic Heart Disease as a global platform to accelerate and monitor its implementation. We affirm the United Nations Declaration on the Rights of Indigenous Peoples (5),^2^ and in particular the rights of Indigenous peoples to determine and administer their own health programmes and to govern data concerning them. We affirm the World Health Organization guidance on the prevention and diagnosis of rheumatic fever and rheumatic heart disease (6), and the principle of universal health coverage.

This Declaration builds directly on the First World Congress in Abu Dhabi in 2023 and on what followed it: the World Heart Federation guidelines for the echocardiographic diagnosis of rheumatic heart disease (7); the establishment of the World Congress as a standing global platform; the expansion of national registers and control programmes; and the subsequent World Health Organization guidance (6). We celebrate that progress. We also plainly record that it has been uneven, under-resourced, and too slow to reach the communities bearing the greatest burden of this disease. Perth is not a starting point. It is an accounting and a commitment to move from evidence to implementation.

Our vision is the worldwide elimination of rheumatic heart disease as a public health problem.

This is achievable through implementing what is already known to work, supported by scientific progress, collaborative research, political will, sustained financing, and health systems grounded in equity.

**A note on what elimination means, and what it does not**. We call for elimination rather than eradication. Eradication means reducing an organism worldwide to zero, and Group A Streptococcus, a common coloniser of the human throat and skin, is not a candidate for it. Elimination means reducing rheumatic fever and rheumatic heart disease to a level at which they are no longer a public health problem, measured against agreed thresholds for incidence, prevalence, morbidity, and mortality. What this requires differs between a country with widespread transmission and no register and one where the disease persists only in particular communities. We state clearly what elimination does not mean. It is a commitment to end new cases and to change the conditions that cause them. It says nothing about the worth, the future, or the place of the many people living with this condition today, to whom we owe the best care available for as long as they need it. We set 2040 as the global target because changing those conditions takes a generation. We welcome and support every country and community that commits to reaching it sooner.

## What We Commit To

These are commitments we make as a community, the people, organisations and researchers gathered in Perth, for the next three years, and on which we will report at the Third World Congress in 2029. Each is anchored in a workshop or special session of this Congress, and each will produce a published action paper setting out what we intend to achieve, who is accountable for it, and how it will be measured.

1. **Partner meaningfully with affected people and communities**.
  We will support community-led and community-controlled action, and ensure that those closest to the disease shape priorities, programmes, policies, research and decisions as partners with authority, not as consultees. We will elevate the voices of young people, and recognise patient support groups, peer networks and community-led events as central to advocacy and partnership rather than adjuncts to it. Where the disease affects Indigenous peoples, community-led approaches, and community-controlled organisations and health services, should be supported and resourced to lead this work.
2. **Act on the conditions that give rise to the disease**.
  We will work with colleagues in housing, water, sanitation, education and environmental health to strengthen the evidence for primordial prevention and to make the case for investment in it. We will strengthen recognition and timely treatment of Group A streptococcal throat and skin infection in primary care, and advance diagnostic capability for acute rheumatic fever so that earlier diagnosis and protective prophylaxis become feasible in the settings where they are most needed.
3. **Develop scalable models for earlier detection**.
  We will develop and test implementation models that expand equitable access to echocardiographic screening, so that earlier diagnosis and protection from progression reach the places where rheumatic heart disease is most common. We will work to reduce cost and complexity so that these models can be adopted quickly and sustained locally, and we will be explicit about the conditions under which screening delivers benefit, so that countries can plan realistically.
4. **Improve care and support for people living with rheumatic heart disease**.
  We will develop and test models that strengthen the delivery of secondary prophylaxis and support people to continue treatment, through community-based approaches, peer support, and initiatives that build on people’s own strengths and circumstances rather than attributing missed doses to them. This includes better tools for monitoring, support networks for families, and work to understand and reduce stigma. We will advocate for the long-term supports that people with established disease require, including anticoagulation monitoring, heart failure care, and psychosocial and wellbeing support.
5. **Place women and girls at the centre of the response**.
  We will ensure that screening, prophylaxis, and treatment are designed around the lives of those most affected, and we will establish better estimates of the contribution of rheumatic heart disease to maternal mortality. We will work to understand and reduce the stigma and social pressures experienced by women and girls living with this condition.
6. **Build data systems that serve communities**.
  We will support registries and monitoring systems that track the reach and impact of interventions and are integrated within national health information systems. These systems will be designed to be scalable and sustainable, to support routine care for individual patients, to record met and unmet needs for cardiac intervention, and to drive quality improvement. Where data concern Indigenous peoples, they will be governed in accordance with the principles of Indigenous data sovereignty (8,9), collected, held, interpreted, and used under the authority of the communities they describe, and returned to them in a form they can use.
7. **Amplify the voices that drive change**.
  We will build advocacy capacity in affected countries and communities so that people living with the disease, clinicians, and community organisations can keep rheumatic heart disease on national and global agendas and hold decision-makers to account.
8. **Hold ourselves accountable**.
  We will report publicly on progress against each of these commitments at the Third World Congress in 2029, drawing on the action papers and monitoring frameworks developed in Perth, and through national and global mechanisms, including World Health Organization reporting channels.

## What We Ask of Governments and National Health Authorities

- **Name it, and fund it for the long term**. Elevate rheumatic heart disease within national health plans and health coverage arrangements, integrated into primary health care and maternal and child health services. Fund national control programmes with sustained, predictable, multi-year investment rather than short cycles of project funding; this disease requires programmes that outlast electoral terms. Set measurable targets, report publicly against them, and increase domestic investment.
- **Invest in the conditions that prevent the disease**. Prioritise environmental health: housing that is not overcrowded, reliable water and power, functioning washing and bathing facilities, and maintenance of that infrastructure over time. Fund this work through housing, water, infrastructure and Indigenous affairs portfolios alongside health, and hold those portfolios jointly accountable for health outcomes. No programme of treatment can outrun the conditions that make it necessary.
- **Resource community-led and community-controlled delivery**. Fund Aboriginal and Torres Strait Islander community-controlled health organisations, and their equivalents among Indigenous peoples and affected communities elsewhere, to design and deliver this work. Support strengths-based models of care that are culturally safe, locally governed and built on the capabilities communities already hold. Programmes designed with communities are more effective than programmes designed for them.
- **Secure the penicillin supply chain**. Work towards uninterrupted supply of affordable, quality-assured benzathine penicillin G, oral penicillin and future delivery systems, through procurement planning, buffer stocks and regulatory efficiency.
- **Improve early diagnosis and treatment**. Ensure community-level access to prompt diagnosis and treatment of Group A streptococcal throat and skin infection, and to timely diagnosis of acute rheumatic fever and rheumatic heart disease. These are distinct tasks requiring distinct capabilities, and national plans should say so.
- **Establish and sustain registers, under appropriate governance**. Support national and community registers as the standard of care: register every person diagnosed, follow them actively, and report programme performance including the proportion of people needing an intervention who receive it within 12 months. Where registers hold data concerning Indigenous peoples, governance must rest with those peoples and their organisations.
- **Build the workforce and the tools**. Invest in workforce training, including training, employment and career pathways for people from affected communities, and in diagnostic capacity, including echocardiography, and digital health tools developed in partnership with the communities they serve.
- **Protect young women**. Integrate detection and management of rheumatic heart disease into antenatal care in affected regions, and ensure safe cardiac care for women of reproductive age, with attention to the specific stigmas they face.
- **Care for those already affected**. Expand regional surgical and interventional cardiology capacity to reduce waiting times, and ensure life-saving procedures are accessible and affordable to those who need them.
- **Prioritise continuity of care during times of crisis**. Support patients and families to continue to access life-saving care during crises such as war and natural disasters.
- **Integrate awareness into education systems**. Support age-appropriate health education in schools and training institutions, so that students and communities can recognise early signs and seek care, and so that stigma is reduced.

## What We Ask of Health Services and Clinical Leaders

- **Make register-based care routine practice**. Ensure every person diagnosed is registered, actively followed, and supported to continue treatment, and that programme performance is reviewed as a matter of course.
- **Build capability in your own workforce**. Train and credential non-specialists in focused echocardiography, prevention and environmental health. Build local surgical and interventional teams capable of caring for patients in their own country.
- **Integrate wellbeing and psychosocial support into care**. Improve access to tailored psychosocial support and treat wellbeing as part of rheumatic heart disease care rather than an optional addition.

## What We Ask of Global Partners

- **Fund implementation of what already works**. The greatest immediate returns lie in delivering existing, proven interventions at scale. Support the development, testing, and spread of implementation models for integrated prevention, diagnosis, and care. Fund learning networks that move knowledge between programmes quickly. Prioritise rheumatic heart disease within health system strengthening and neglected disease financing.
- **Improve access to penicillin**. Strengthen the benzathine penicillin G supply chain, diversified and additional manufacturing capacity, pooled and regional procurement, regulatory harmonisation and price transparency, and invest in new formulations and delivery systems together with the evidence for their use.
- **Sustain research that answers the questions communities are asking**. Fund clinical trials and innovation in rheumatic heart disease, weighted towards implementation and health systems research led by institutions in affected countries and shaped by community priorities. Require that international research partnerships confer authorship, capacity, infrastructure and data sovereignty on the countries and communities in which the research is conducted.
- **Agree the regulatory pathway for a vaccine, and then finance the trials**. Regulators have not agreed on a pathway to licensure for Group A streptococcal vaccines, and there is no agreed-upon guidance for assessing vaccine-induced autoimmunity in preclinical and clinical studies. This is a rate-limiting step that financing alone will not resolve, and it has a history: a flawed trial in the 1960s and the regulatory overreaction that followed it held the field back for a generation (10).^3^ The tools now exist to understand, minimise and assess that risk. We call on national regulatory authorities, World Health Organization prequalification and the regional regulatory networks to publish an agreed pathway, including autoimmunity assessment guidance, by 2028; on funders to finance phase 3 evaluation to commence by 2031, developed in accordance with the World Health Organization Preferred Product Characteristics and Research and Development Technology Roadmap (11); and on all partners to plan now for rollout to the populations most in need by 2040 (12). A vaccine will not replace the other priorities in this Declaration; instead, it will provide a critical additional tool for tackling rheumatic heart disease.
- **Frame elimination as an investment**. Treat elimination as a sound economic investment in human capital as well as a matter of equity. Rheumatic heart disease affects people during the years of education, family life and work, and continues to cause avoidable deaths. Prevention costs a fraction of surgery, long-term care and lost livelihoods.

## What Has, and Has Not, Been Achieved

This Declaration presents itself as an accounting. An accounting begins with the last set of commitments.

In 2013, the World Heart Federation set a target of a 25% reduction in premature deaths from rheumatic fever and rheumatic heart disease in people under 25 years by 2025 (13). We report the following. On Global Burden of Disease 2021 estimates (14),^4^ the age-standardised death rate among children and adolescents aged 5–19 fell from 1.23 to 0.52 per 100,000 between 1990 and 2021, an average of 2.7% a year; extrapolated to 2025, that trend is consistent with the reduction of the order the target sought. Three qualifications are necessary. The fall is in age-standardised rates rather than absolute deaths, which have declined far less against population growth, and it has been uneven: mortality has fallen fastest where health systems are strongest, and least in sub-Saharan Africa and South Asia. Over the same period age-standardised prevalence in this age group rose by about 18% and incidence by about 20%, so this is not evidence of reduced transmission. And in most endemic countries, no surveillance system exists that could verify any of it; the assessment rests on modelled estimates rather than counted deaths.

We therefore report the 2013 target as not verifiable from surveillance data, and as probably approached on modelled estimates. We regard our inability to answer that question properly as the most consequential finding of the period, and it is the reason Commitment 6 exists.

Since the adoption of the World Health Assembly Resolution in 2018, and more recently since the First World Congress in Abu Dhabi in 2023, we record: the publication of the World Heart Federation guidelines for the echocardiographic diagnosis of rheumatic heart disease in 2023 (7); the World Health Organization guideline on the prevention and diagnosis of rheumatic fever and rheumatic heart disease in 2024 (6); and the establishment of the World Congress as a standing platform. On registers, the World Health Organization’s country capacity surveys (15,16) provide a pre- and post-resolution comparison: 28% of the 74 countries where rheumatic heart disease is endemic had a register in 2023, against 27% in 2017; four in five of those registers had a system for follow-up or recall. No country is currently known to operate a comprehensive national control programme as this Declaration defines one. Against those, we record no measurable global change in age-standardised prevalence or incidence, and no published global figure for secondary prophylaxis coverage. Those absences are themselves findings.

## Targets

We will judge our efforts by the progress that follows this Declaration. Our targets are phased. The first set of targets are for the rheumatic heart disease community to deliver. Subsequent targets are benchmarks against which to measure the responsiveness of governments, health systems and global partners.

Unless otherwise stated, mortality and burden targets are measured against a 2025 baseline, using national vital registration and register data where available and Global Burden of Disease estimates where they are not.

We state plainly that the thresholds against which elimination will be judged, and the agreed baselines for several targets in this Declaration, do not yet exist. That is why the first deliverable of this Declaration is the technical paper that will define them, and why we have brought it forward to within 12 months of this Congress. Until that paper is published, the 2035, 2036 and 2040 targets will serve as an interim reference against Global Burden of Disease estimates for 2025 and will be restated against the agreed baselines upon publication. The paper will be peer-reviewed and openly published, and the baselines it sets will not be revised retrospectively once progress against them is known.

**By 2027, within 12 months of this Congress:**

- A technical paper, peer-reviewed and openly published, defining the thresholds for incidence, prevalence, morbidity and mortality against which elimination will be measured; the baselines and data sources for every target in this Declaration; and the context-specific pathways to reach them.

**By 2028:**

- An agreed regulatory pathway to licensure for Group A streptococcal vaccines, published by national regulatory authorities together with the World Health Organization, and including agreed guidance on the pre-clinical and clinical assessment of vaccine-induced autoimmunity before and during first-in-human studies.

**By 2029, at the Third World Congress, we will report on:**

- A published action paper for each of the eight commitments above, each with a named accountable lead, a small set of measurable indicators agreed by the community, and a public account of progress against them.
- A community-agreed core set of indicators, a rheumatic heart disease care cascade, against which programmes can benchmark and improve the care they deliver.
- A participation standard adopted across Congress-affiliated work, reported as the proportion of publications, guidelines, and funded studies with lived-experience co-authorship or co-investigators.
- A shared rheumatic heart disease resource hub, with utilisation tracking, that can serve to increase access to tools to promote public awareness and education to improve understanding of rheumatic heart disease, reduce stigma, encourage early diagnosis and treatment, and empower communities to participate actively in prevention and care.

**By 2031:**

- At least one Group A streptococcal vaccine candidate entering phase 3 evaluation, with the trial fully financed and trial capacity established in affected countries.

**By 2032, as benchmarks for governments, health systems, and global partners:**

- Measurable year-on-year improvement in the reliability of benzathine penicillin G supply in reporting countries.
- A funded, functioning national programme with an operational register in a growing majority of affected countries.
- Measurable increases in equitable access to cardiac surgery and intervention and in the coverage and continuity of secondary prophylaxis.

**By 2035:**

- A reduction of at least 50% in mortality from rheumatic fever and rheumatic heart disease among people under 25 years, against a 2025 baseline, in countries with established surveillance, doubling the ambition of the World Heart Federation target set in 2013, on the same measure.

**By 2036:**

- Licensure of a safe and effective Group A streptococcal vaccine. This target is conditional on phase 3 evaluation commencing by 2031 with full financing secured; on the intervals currently estimated for phase 3, bridging studies and regulatory review, licensure is not achievable by 2036 on any later start.

**By 2040:**

- Rheumatic fever and rheumatic heart disease eliminated as a public health problem, against agreed thresholds for incidence, prevalence, morbidity and mortality, with countries and communities that have set earlier dates supported to reach them.
- A safe and effective Group A streptococcal vaccine available and accessible at an affordable price to the people most in need, under advance access and pricing commitments agreed at the time of licensure rather than negotiated after it.

The World Heart Federation and the signatories to this Declaration will track progress against these targets and report publicly at future World Congresses, and through national and global monitoring frameworks, including World Health Organization mechanisms.

## From Perth

That rheumatic heart disease survives into the present is not a mystery of medicine. It is a decision, taken by default, renewed by inattention, and reversible by will. We know what needs to be done, and we know that it can be done. Today we decide to change the future of this disease. We choose equity. We choose coordinated action. We commit to doing our part, and we call on those with the power to deliver the rest to do theirs.

**Together, we will #EliminateRHD**

## Perth Declaration Writing Group

- Prof. Jagat Narula – President, World Heart Federation
- Prof. Garry Jennings – Chief Medical Advisor, National Heart Foundation of Australia
- Prof. Andrea Beaton – Associate Professor of Paediatrics, Cincinnati Children’s Hospital
- Prof. Liesl Zühlke – Vice-President, South African Medical Research Council
- Prof. Jonathan Carapetis – Co-Head, Strep A Translation Team, The Kids Research Institute Australia and University of Western Australia
- Prof. R. Krishna Kumar – Amrita Institute of Medical Sciences and Research Centre; Chair of the Neglected Cardiac Diseases Expert Group, World Heart Federation
- Dr Dawn Casey – CEO, National Aboriginal Community Controlled Health Organisation
- Dr Ben Jones – Early career clinician scientist, The Royal Children’s Hospital and Menzies School of Health Research
- Dr James Marangou – Consultant cardiologist, Royal Perth Hospital and Menzies School of Health Research
- Dr Bente Mikkelsen – Chair, Advocacy Committee, World Heart Federation
- Prof. Maria Do Carmo Nunes – Full Professor, Federal University of Minas Gerais and Head of Echocardiography Laboratory, Hospital das Clinicas
- Dr George A. Mensah – Director of the Center for Translation Research and Implementation Science, National Heart, Lung and Blood Institute
- Prof. Dejuma Yadeta – Chair, Pan African Society of Cardiology RHD Taskforce
- Erini Tokarua – Global Lived Experience Experts Group
- Anu Gomanju – Global Lived Experience Experts Group
- Christine Katusiime – Lived Experience Patient Advocate
- Ronnie Odak – Lived Experience Patient Advocate
- Zoë Fitzgerald – RHD Policy and Program Lead, National Heart Foundation of Australia
- Prof. Derek P. Chew – Chief Scientific and Medical Officer, National Heart Foundation of Australia

## Perth Declaration Endorsing Organisations (as at 22 september 2026)

- World Heart Federation
- National Heart Foundation of Australia
- Reach
- National Aboriginal Community Controlled Health Organisation

## Data Availability

This article reports no new data. All estimates are drawn from the published sources cited.

## Footnotes

Reference (1). Global Burden of Disease 2021 estimates for 2021: 54.8 million prevalent cases (95% uncertainty interval [UI] 43.3–67.6 million) and 373,345 deaths (95% UI 324,140–444,762); age-standardised prevalence rate +12.6% and age-standardised death rate –56.2% between 1990 and 2021.

Reference (5). In particular Articles 23 and 24, on the right to determine and administer health programmes, and Article 31, on control of cultural knowledge and data.

Reference (10). The Massell trial (1965–1967); 21 CFR 610.19, codified in 1979 and revoked on 2 December 2005; and the absence of regulatory consensus on pre-clinical assessment of vaccine-induced autoimmunity before first-in-human studies.

Reference (14). Ages 5–19 years, 1990–2021: age-standardised death rate 1.23 to 0.52 per 100,000 (estimated annual percentage change –2.71%); age-standardised prevalence rate 498.49 to 588.46 per 100,000 (+18.0%); age-standardised incidence rate 77.98 to 93.96 per 100,000 (+20.5%).

## Use of generative AI tools

Generative AI was used in preparing this submission. Claude (Anthropic; Claude Opus 5.5) was used to check grammar, number style, quotation style, in-text citations and the reference list to Global Heart requirements, and to check bibliographic details against PubMed. All AI-assisted changes were tracked changes and reviewed, edited and approved by the authors, who take full responsibility for the content. Generative AI was not used to generate the commitments, asks or targets of the Declaration, which were developed by the Writing Group and adopted by the Congress.
